# Forecasting Surgical Bed Utilization: Architectural Design of a Machine Learning Pipeline Incorporating Predicted Length of Stay and Surgical Volume

**DOI:** 10.1007/s10916-025-02201-3

**Published:** 2025-05-21

**Authors:** Arjun Singh, Patrick E. Farmer, Jeffrey L. Tully, Ruth S. Waterman, Rodney A. Gabriel

**Affiliations:** 1 Division of Perioperative Informatics, Department of Anesthesiology, University of California, San Diego, La Jolla, San Diego, CA USA; 2 Department of Biomedical Informatics, University of California, San Diego Health, La Jolla, San Diego, CA USA

**Keywords:** Perioperative, Bed utilization, Artificial intelligence, Machine learning

## Abstract

The objective of this study was to develop a machine learning model utilizing data from the electronic health record (EHR) to model length of stay and daily surgical volume, in order to subsequently predict daily surgical inpatient bed utilization. Machine learning is increasingly used to aid healthcare decision-making and resource allocation. Surgical inpatient bed utilization is a key metric of hospital efficiency and an ideal target for optimization. EHR data from all surgical cases over one year at a single institution was obtained. Data from the first 32 weeks of the year were used to train the model with the remaining data used to validate and test the models. Various machine learning approaches were explored to predict hospital length of stay and surgical volume. Seasonal Autoregressive Integrated Moving Average (SARIMA) was used to forecast daily surgical bed requirements. The root mean squared error (RMSE) was reported. For predicting bed utilization > 2 weeks in the future, our optimized models improved prediction from an RMSE of 43.1 to 24.4 beds. For predicting bed utilization in 2 weeks, our optimized models improved prediction from an RMSE of 42.6 to 24.8 beds. Finally, predicting bed utilization same day demonstrated an RMSE of 22.7 beds. We described the architecture of a machine learning approach to forecast surgical bed utilization. Forecasting use of surgical resources may decrease stress on a hospital system through more accurate predicting of the ebbs and flows of hospital needs.

## Introduction

The daily volume of elective surgical patients requiring a planned postoperative admission is often a predictable quantity. However, unexpected daily occurrences that are a challenge to foresee may also affect surgical bed utilization, including emergent procedures or unplanned admissions following outpatient surgery. This is especially true for trauma centers or healthcare institutions that provide surgical services for medically complex patients, where routine cases may be followed by exacerbations of existing comorbidities [1]. While bed availability is critical for ensuring adequate access to care, there are also significant financial implications in optimizing operating room scheduling based on trends with inpatient surgical bed availability [2, 3]. Under- or over-utilization of surgical bed capacity may correspond to loss of potential revenue and may also be associated with patient safety impacts [4–9].

Having the ability to forecast daily surgical inpatient bed utilization may aid in optimizing elective operating room scheduling. There are various clinical variables that may contribute to the required daily bed capacity for surgical patients, including the number of elective inpatient surgeries, emergent and/or urgent surgeries, unplanned admissions following outpatient surgery, hospital admissions for non-surgical patients, hospital length of stay, and unexpected same-day surgical cancellations [10]. Patterns of daily surgical bed utilization may be elicited using machine learning approaches. Novel machine learning-based approaches have been previously described to solve challenges in healthcare resource allocation and quality improvement [11–13].

The purpose of this study is to develop machine learning models to predict future inpatient surgical bed requirements based on historic surgical volume trends at a single institution. This may allow for proper planning of patient load to ensure appropriate space for post-surgical patients and assist in optimizing staffing. This type of model would be specific to each institution and should have minimal error in order to facilitate strategic decisions regarding operating room management.

## Methods

### Study Sample

This study and the associated collection of data from our electronic medical record system was approved by the University of California San Diego’s Human Research Protections Program and the requirement for informed consent was waived. In this retrospective study, data from all surgical/procedural encounters at this institution from July 2022 to June 2023 were extracted from the electronic health record database.

### Study Objectives

**Fig. 1:**
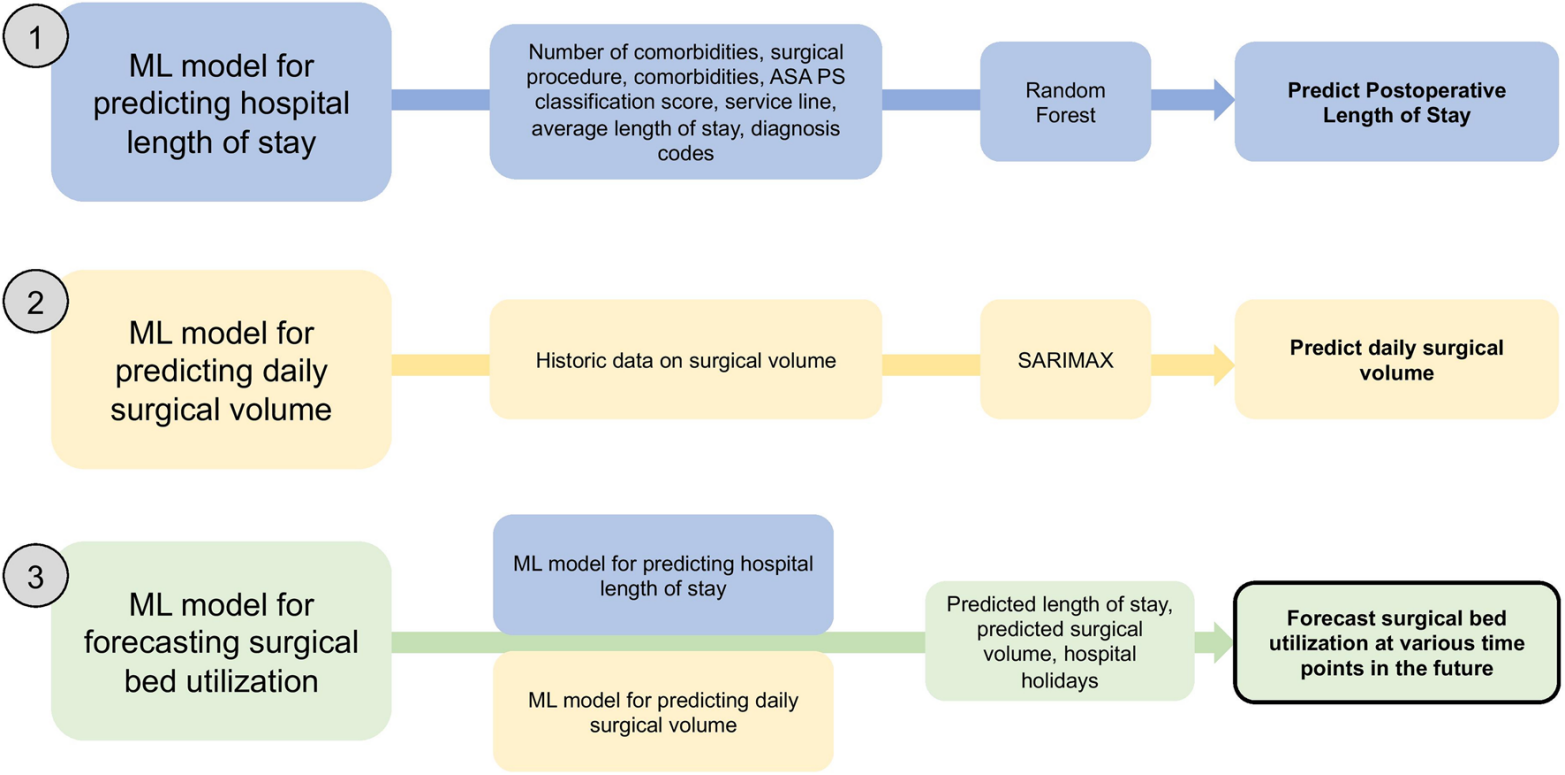
Schematic illustration of machine learning approach to: (1) predicting postoperative hospital length of stay; (2) predicting surgical volume on a given day; and (3) forecasting surgical inpatient bed utilization on a given day in the future. Abbreviations: ML, machine learning; SARIMAX, seasonal autoregressive integrated moving average

The objective of this study was to develop time series machine learning models that may forecast surgical bed utilization at various points in the future. We first developed two separate predictive models that may drive surgical inpatient bed utilization: (1) estimated hospital length of stay after a given surgery; and (2) estimated total number of surgical procedures to be performed on a given day. We then incorporated the predictions from these models to then construct a machine learning model that may forecast inpatient surgical bed utilization in the future (Fig. 1). We focused on three future time points for forecasting bed utilization: (1) ≥ 2 weeks in the future; (2) 2 weeks into the future; and (3) same day (prior to start of operating room day).

### Description and Preprocessing of Dataset

We acquired retrospective data from a single institution derived from all surgeries performed during a one-year time span. For each surgical encounter, the following data points were obtained from each patient: (1) comprehensive list of patient comorbidities (International Classification of Disease, 9th Revision [ICD-9] and 10th Revision [ICD-10] codes) and total number of comorbidities; (2) age; (3) surgical procedure; (4) surgical service line; (5) sex assigned at birth; (6) urgency of procedure (e.g., elective, same-day add-on, urgent [must be performed in 6 h], or emergent [must be performed in 1 h]); (7) scheduled surgical case duration (minutes); (8) American Society of Anesthesiologists Physical Status (ASA PS) score; and (9) day of the year (to model data as a time series).

Patient records missing the type of surgical procedure were removed. Missing data for scheduled case duration and patient length of stay were imputed by taking the average of the respective value from other patients undergoing the same type of surgery. Missing data for ASA PS scores were imputed by taking the mode for each surgery type. If all values of a specific surgery were missing, the features for that surgery were assigned a value of “unknown.” For patient length of stay, missing values were imputed by the calculating the mean for that surgery. There were no singular cases with missing data that would require imputation. There were no missing values for scheduled case duration. Next, we determined which comorbidities based on ICD9/10 codes were to be included in the analyses. There were 57,680 unique comorbidities. Each patient had an average of 10.9 (standard deviation = 11.2) with a range of 1 to 160 associated comorbidities. One-hot encoding all the comorbidities would not be feasible due to the exponential memory cost. Furthermore, encoding this many features would be computationally expensive and risk overfitting for a dataset of ≥ 70,000 records. Thus, we chose to utilize the top *n* most frequent comorbidities as most comorbidities occurred infrequently. Solving for *n* was a matter of hyperparameter tuning against model performance. Thus, we assessed model performance for hospital length of stay (based on mean squared error [MSE]) based on the number of comorbidities included as mode inputs. After selecting the top 1,750 most frequently occurring comorbidities, the resulting matrix was sparse and relatively large [14]. Zeroes made up 99.6% of the elements in the matrix. By applying some decomposition or feature agglomeration method, the comorbidity matrix size was further reduced by two orders of magnitude. The number of elements used to train the model reduced to approximately 340,000 elements from 42 million elements. Principal component analysis, truncated singular value decomposition, and feature agglomeration were first tuned to the optimal number of components and then compared by evaluating downstream model loss. This may help manage memory costs, improve training speed, and reduce overfitting.

### Statistical Analysis and Machine Learning Architecture

Python (v3.7.5) was used for all machine learning approaches. The machine learning approaches for each predictive model (hospital length of stay, surgical volume, and bed utilization) are described below. For each model, the dataset was split into training: validation: test sets. Data before the 32nd week (225 days) was used for training, the next 70 days were used for validation, and the last 70 days were used for testing. When testing, the best model(s) was retrained using the combined training and validation data and evaluated using the hold-out method to test the data.

The first task was to develop machine learning models for predicting hospital length of stay for a patient undergoing surgery. The features included in this model included number of comorbidities, the 12-component decomposed comorbidities (as described above), surgical service line (the department plus inpatient vs. outpatient designation), ASA PS score, scheduled case duration, and an engineered feature (the historic average length of stay for that surgery type). The comorbidity training set only consisted of comorbidities observed in the first 32 weeks. Thus, the validation and test sets were filtered to contain only the comorbidities occurring in the training comorbidity set. Regression (using LASSO), support vector machine (SVM), decision tree, random forest, and gradient boosting random forest (XGBoost) algorithms were initially evaluated. However, linear regression failed to converge likely due to the complexity of inputs while the decision tree algorithm demonstrated large fluctuations in MSE from interval changes in dataset training windows (which could indicate limitations with a single decision tree model.) LASSO regression and SVM also yielded inferior results. While the XGBoost implementation of gradient boosting random forest had better overall RMSE than the regular random forest, the gradient boosting model performed worse for patients with a length of stay less than 10 days, which accounts for 93% of the dataset. Thus, random forest was the optimal machine learning approach for this prediction task. Random forest is an ensemble technique that combines the predictions from multiple machine learning algorithms together to make more accurate predictions than any individual model [15]. Random forest is a robust and reliable non-parametric supervised learning algorithm that tests further improvement in metrics and demonstrates the importance of a feature in the dataset. Several hyperparameters were tuned including tree maximum depth, maximum number of leaf nodes, minimum samples per split, number of trees, and number of samples assigned to each tree. Maximum number of leaf nodes and the number of samples per tree had the greatest impact on model performance.

The second task was forecasting surgery volume (i.e. expected number of surgeries to occur in a given day). Our primary machine learning approach to forecasting daily surgical count was Seasonal Autoregressive Integrated Moving Average (SARIMA), which is a technique for forecasting time series data using a combination of autoregressive models and moving average models [16]. SARIMA is especially useful when modeling data that has seasonality patterns, such as that seen with operating room scheduling, in which surgical volume depends on a typical work week (five weekdays and 2 weekend day) and scheduled holidays. The notation for SARIMA is SARIMA (p, d,q)(P, D,Q)m + exogenous variable, which describes its hyperparameters, where.

p = trend autoregression order,

d = trend difference order,

q = trend moving average order,

P = seasonal autoregressive order,

P = seasonal autoregressive order,

D = seasonal difference order.

Q = seasonal moving average order.

m = the number of time steps for a single seasonal period.

Of note, the term SARIMAX is used when an exogenous variable is used for the predictive model. To determine the optimal hyperparameters, we calculated the autocorrelation function (ACF) (to determine the correlation between values of the signal changes as their separation changes) and partial autocorrelation function (PACF) (which measures the correlation between observations of a time series that are separated by a specified time after adjusting for the presence of all the other terms of shorter lag) [17]. Several SARIMAX models were fit to the daily counts of surgeries from the training set and forecasted for on the test set. The best model was further evaluated for generating rolling, one-week-ahead forecasts. Daily counts of surgeries and hospital holidays were exogenous features included in predicting the number of surgeries on a given day.

Finally, we developed a model to predict inpatient surgical bed utilization at a given time point. Features that were included to forecast surgical bed utilization were daily count of surgeries, observed overnight stays, predicted overnight stays, and hospital holidays. Predicted overnight stays were calculated by individually predicting the length of stay for each patient via random forest (as described above) and then aggregating the estimated total number of patients staying overnight. The surgery dataset was used to derive the number of patients staying overnight which is then populated in an array representing each day. A similar array was constructed using the predictions from the random forest regressor. The fit of multiple SARIMA models was evaluated. Some models were further evaluated for their ability to make rolling predictions on various forecast windows into the future. As a comparison, Viability of Ordinary Least Squares Regression [18] and Vector Autoregression [19] were also assessed. The metrics used to measure performance for all models were the root mean squared error (RMSE) and its standard deviation (SD) (Fig. 1).

## Results

### Study Population

The initial total number of surgeries was 75,283. One record missing the type of surgery conducted was removed. After exclusion, the final study population contained 75,282 surgeries for 49,682 unique patients spanning 57,680 unique diagnosis codes. There were 86 cases with missing length of stay data and thus duration was imputed based on the average length of stay for the corresponding surgery. The median (quartiles) age of patients was 57 (45, 70) years old (Table 1). There were 54,492 (72.4%) surgical encounters classified as an outpatient procedure. There were 1,866 unique types of surgeries, with the four most common procedures (23.6% of records) being colonoscopy, esophagogastroduodenoscopy, phacoemulsification and cataract extraction, and cesarean delivery. The median (quartiles) hospital length of stay was 1 (1, 2) day.

**Table 1:** Distribution of baseline data

Variable	Distribution
Age (years), median [quartile]	60 [45, 70]
Male sex, n (%)	46.420%
ASA PS, median [quartile]	3 [2, 3]
Scheduled case duration, median [quartile]	55 [30, 105]
Hospital length of stay (days), median [quartile]	1 [1, 2]

Abbreviations: ASA PS, American Society of Anesthesiologists Physical Status

### Predicting Hospital Length of Stay after Surgery

As there were 57,680 possible diagnosis codes represented in this population, we performed dimensionality reduction utilizing truncated support value decomposition and feature agglomeration versus principal component analysis. The best comorbidity decomposition method was truncated support value decomposition, which identified 12 components applied to the top 1,750 frequently occurring comorbidities. Feature agglomeration failed to reduce downstream loss. Truncated support value decomposition resulted in the lowest downstream loss and marginally outperformed principal component analysis.

Next, we developed a random forest model to predict postoperative hospital length of stay, which incorporated the following features: age, sex, comorbidity components (from dimensionality reduction), average historic length of stay, surgical service line, ASA PS classification score, scheduled case duration, and surgical procedure. The best random forest used a maximum of 92% of the training set per tree, and a maximum of 580 leaves per tree. The RMSE was 5.02 days and the mean absolute error was 1.769. Out of the 35 features (after one-hot encoding) the 5 most important features in order based on permutation feature importance are planned inpatient admission, mean length of stay for that surgery, planned hospital outpatient procedure, and 2 singular value decomposition features. Among the top 10 important features, 4 of the 12 components of the singular decomposition of comorbidities were included.

### Predicting Surgical Count Per Day

**Fig. 2:**
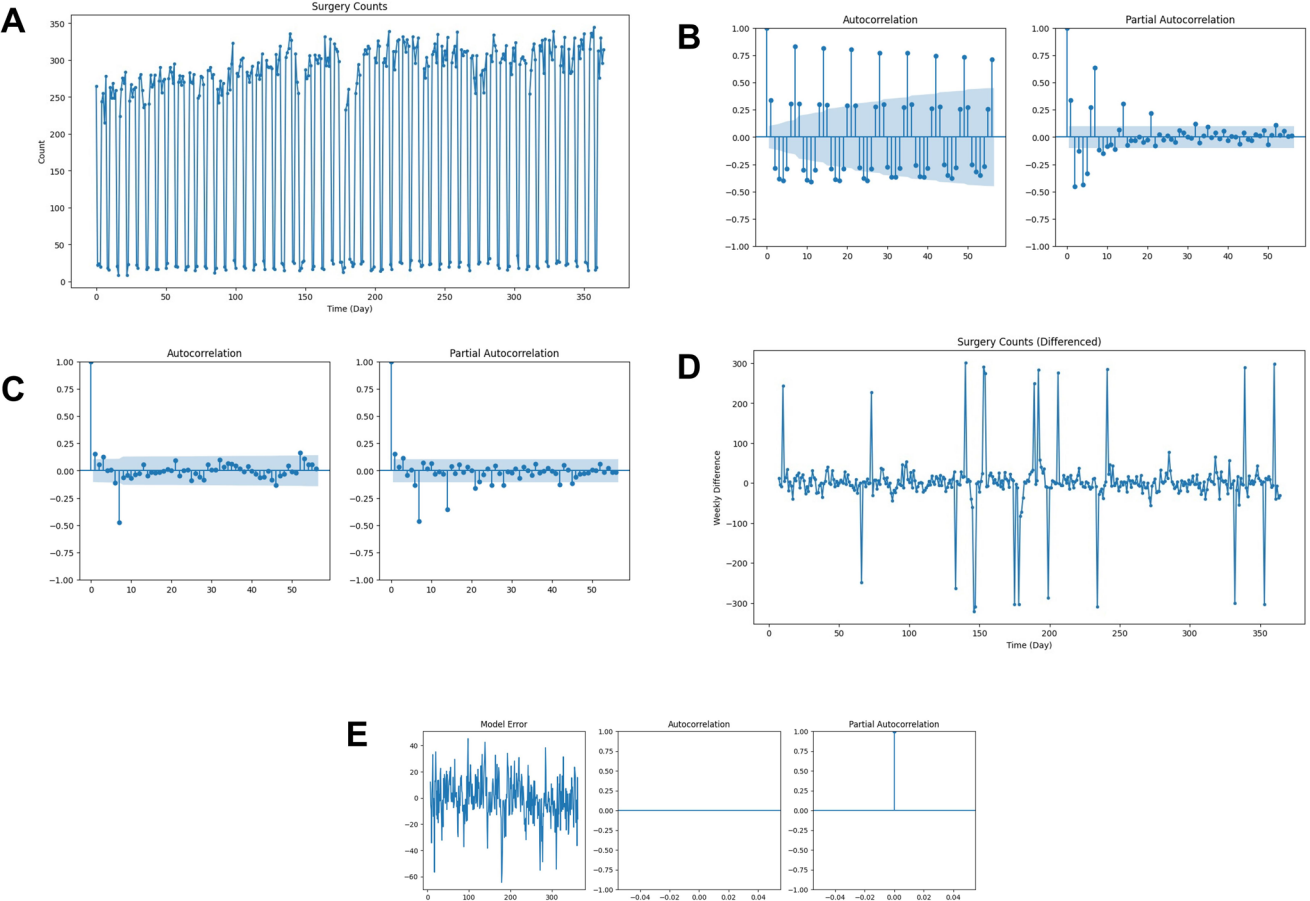
**A**) daily surgical counts on a given day from day 0 to day 365 in a year; **B**) the calculated autocorrelation function and partial autocorrelation function of daily surgical volume, which indicated the seasonal order was a multiple of 7; **C**) After taking the first order difference between every 7th day, the plots were examined for outlying trends. The partial autocorrelation function was diminished every 7th lag, whereas the autocorrelation function had a single significant value at the 7th lag, which suggested a seasonal moving average term of 1; **D**) The 1st order 7th day difference of daily surgery counts visually contained spikes, which indicated disparities in surgery counts from week to week. Further investigation indicated these were due to hospital holidays; and **E**) inclusion of holidays as an exogenous variable for the model demonstrating elimination of autocorrelation

The median number of surgeries/procedures per day was 278. Daily surgical volume during the study year is illustrated in Fig. 2A. To predict daily surgical volume, we utilized the SARIMA algorithm. The first step in fitting a SARIMA is to determine presence of seasonal order. The autocorrelation function and partial autocorrelation function confirmed the seasonal order was a multiple 7 (Fig. 2B). After taking the first order difference between every 7th day, the plots were examined for outlying trends. The partial autocorrelation function diminished every 7th lag, whereas the autocorrelation function had a single significant value at the 7th lag, which suggested a seasonal moving average term of 1 (Fig. 2C). The 1st order 7th day difference of daily surgery counts visually displayed spikes (Fig. 2D), which indicated disparities in surgery counts from week to week. Further investigation indicated these were due to hospital holidays. Thus, by including holidays as an exogenous variable, autocorrelation issues were eliminated from the model. The model error appeared normally distributed and there were no outstanding significant autocorrelation function or partial autocorrelation function lag terms thereafter (Fig. 2E).

**Fig. 3:**
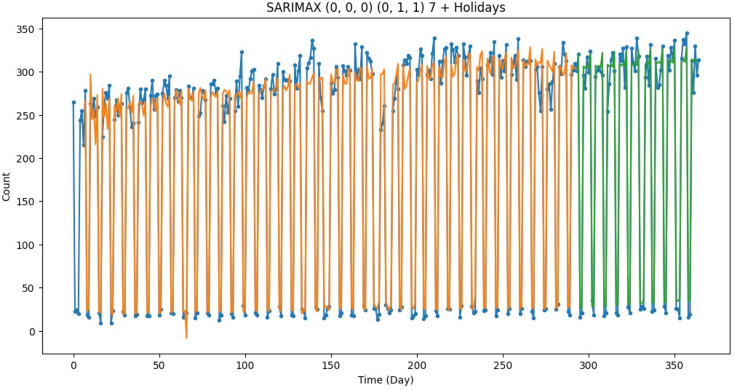
Illustration of actual versus predicted daily surgical volume based on day of the year (day 0 to 365). The blue dots indicated actual surgical volume. The orange dots indicated predicted surgical volume within the training set. The green dots indicated predicted surgical volume within the test set. The model used is SARIMAX (0,0,0)(0,1,1) 7 + holidays, whereby the notation for SARIMAX is SARIMAX (p, d,q)(P, D,Q)m + exogenous variable. Where p = trend autoregression order, d = trend difference order, q = trend moving average order, P = seasonal autoregressive order, P = seasonal autoregressive order, D = seasonal difference order, Q = seasonal moving average order, and m = the number of time steps for a single seasonal period. The exogenous variable included in the model was holidays. Abbreviations: SARIMAX, seasonal autoregressive integrated moving average

The best machine learning model at predicting surgical count per day was a rolling 1-week-ahead SARIMAX with a 7 day seasonality, first order seasonal difference, seasonal moving average term of 1, and an exogenous variable of hospital holidays. The model yielded an RMSE of 15.69 on the test set (Fig. 3).

### Forecasting Inpatient Surgical Bed Utilization

The next step leveraged the predictive models for hospital length of stay and daily surgical volume to forecast surgical bed utilization in the future. We focused on three time points: (1) > 2 weeks into the future; (2) 2 weeks into the future; and (3) same day. Predicted outcomes from the length of stay and surgical volume models were incorporated into this final model. Three machine learning algorithms were assessed, including ordinary least squares regression, vector autoregression, and SARIMAX. RMSE of each model for each time point are listed in Table 2.

**Table 2:** Performance metrics of machine learning models for forecasting inpatient surgical bed utilization at various time points: (1) more than 2 weeks in the future; (2) 2 weeks in the future; and (3) same day

Forecasting Time Point	Machine Learning Model	Exogenous Variable	RMSE	SD
> 2 weeks				
	SARIMA (0,0,0)(0,1,1)7	none	43.08	35.04
	SARIMAX (0,0,0)(0,1,1)7	Holidays	24.38	15.39
	VAR	Holidays, surgery counts	37.37	22.73
2 weeks				
	OLS	Predicted length of stay	42.59	24.71
	SARIMAX (0,0,0)(1,1,0)7	Predicted length of stay	27.69	15.63
	SARIMAX (0,0,0)(1,1,0)7	Holidays, surgery counts	24.77	14.88
Same day				
	SARIMAX (0,0,0)(0,1,1)7	Holidays	25.91	15.92
	SARIMAX (0,0,0)(1,1,0)7	Predicted length of stay	24.85	14.85
	SARIMAX (0,0,0)(1,1,0)7	Predicted length of stay (adjusted)	22.67	13.52

SARIMAX notation

SARIMAX (p, d,q)(P, D,Q)m

p = trend autoregression order

d = trend difference order

q = trend moving average order

P = seasonal autoregressive order

D = seasonal difference order

Q = seasonal moving average order

m = the number of time steps for a single seasonal period

Abbreviations: OLS, ordinary least squares regression RMSE, root mean squared error SARIMAX, seasonal autoregressive integrated moving average SD, standard deviation VAR, vector autoregression

The best long-term (> 2 weeks ahead) forecasting model was the SARIMAX(0,0,0)(0,1,1)7 + Holidays, which demonstrated a RMSE of 24.38. The SARIMAX(0,0,0)(0,1,1)7 + Holidays + 7-Day Lagged Surgery Count performed the best for the 2-week-ahead forecast with a RMSE of 24.77. The RMSE was only slightly better than the SARIMAX(0,0,0)(0,1,1)7 + Holidays. SARIMAX(0,0,0)(1,1,0)7 + Surgery Count + Predicted Length of Stay performed the best for predicting inpatient surgical bed utilization the day of surgery with a RMSE of 24.22. Incorporation of holidays as an exogenous feature did not improve the model for same day forecasting.

The predictions were calculated with the assumption that the hospital staff and model were not aware if a patient will be planned to be discharged the day of surgery. When the prediction array was re-calculated so that the model contained information if a patient will be discharged that day (e.g., planned same day discharge), the forecasts improved even further (adjusted length of stay). The RMSE decreased to 22.67. For reference, the maximum number of inpatients on any given day was 839 across the institution. The true number of beds would be higher than this, implying an average bed utilization prediction error of at most 2.4%.

To study the predictive power of the time series models independent of the upstream length of stay predictions, length of stay was replaced by two similar features: perfect length of stay (the actual length of stay for a case) and average length of stay based on surgery. These two scenarios give a sense for the lower and upper bound prediction error of the models. It was already shown that a model that does not depend on length of stay produced a better 2 week forecast. This remained true even with perfect length of stay information. For same day predictions, perfect length of stay caused the RMSE to decrease to 8.35. Mean length of stay by surgery caused the RMSE to increase to 43.89. An improvement in length of stay predictions could have a demonstratable effect on short term hospital bed utilization forecasts.

## Discussion

In our analysis, we described the architecture of a machine learning approach to forecast surgical bed utilization for an institution. This was done by developing two separate machine learning models (that predicted postoperative hospital length of stay and daily surgical volume) that were then leveraged to forecast bed utilization for surgical patients in the future. For predicting bed utilization > 2 weeks in the future, our optimized models improved prediction from an RMSE of 43.08 to 24.38 beds. For predicting bed utilization in 2 weeks, our optimized models improved prediction from an RMSE of 42.59 to 24.77 beds. Finally, predicting bed utilization same day demonstrated an RMSE of 22.67 beds. Due to several unknown and unpredictable events that may contribute to surgical bed utilization in the future, it would be challenging to develop highly accurate models with near zero RMSE. However, adopting a machine learning framework as described in this study may help hospital administrators in better anticipating needed resources or optimizing operating room scheduling at future time points.

Bed use after surgery can be a confounding factor for the optimal utilization of hospital resources in the perioperative area. Hospitals have an interest in optimizing the use of hospital beds yet this can be a challenging aspect of surgical planning. Therefore, it is crucial to be prepared for the inevitable fluctuations in demand that will occur within any hospital system. Increased perioperative efficiency may improve patient satisfaction scores and decrease bottlenecks that may impact patient care [20]. The use of machine learning has the potential to improve prediction of both surgical volume and length of stay for a given day.

To develop a time series forecasting model for bed utilization, we initially built models that predicted daily surgical volume and post-surgical hospital length of stay. We demonstrated that, in addition to average hospital length of stay, commonly occurring comorbidities may help improve prediction of length of stay. We chose the most frequent 1,750 comorbidities and used the best random forest algorithm to show that length of stay can reasonably be predicted. Although this model was not valid when the expected length of stay is 0 it may improve planning of future post-operative bed needs when surgeries are associated with longer lengths of stay.

Daily counts of surgeries can be also effectively forecasted. SARIMA showed that cases clearly follow a 7-day pattern with good ACF and PACF, which demonstrates increased predictive accuracy with inclusion of holidays as an exogenous variable. We hypothesize that the model can increase in accuracy as more data is included in the dataset. The SARIMAX autocorrelation and partial autocorrelation responses are significant indicators that surgical counts per day and bed utilization are highly predictable at a weekly or bi-weekly rate. Changes in staffing and equipment needed for increases or decreases in surgical volume may not be implemented instantaneously but rather may require time to ramp up or down. Administrators utilizing such models may thus be capable of proactively optimizing staffing or equipment to account for these shifts. Length of stay and daily surgery volume can help forecast inpatient surgical bed utilization with potentially acceptable error. This may increase efficiency and reduce waste in the hospital setting.

Previous studies have attempted to solve various parts of this pipeline [21–23]. For example, some studies have described methods to predict length of stay using patient data similar to what was used in this study. One such study built a 2-stage system for classifying if the predicted length of stay was greater than one week and then estimating the length of stay using a random forest [24]. Samples with prolonged length of stay (> 35 days) were omitted from that training set. They achieved an mean absolute error of 1.73 days. Interestingly, this dataset contained only 16 comorbidities and included medication data. The study concluded that random forest was the most robust model, just as we showed in our study. Another study developed a technique for forecasting inpatient bed demands [25]. They combined K-Means clustering with support vector machine regression to forecast inpatient bed demand without the need for sensitive patient data. The models achieved a mean absolute percentage error of 1.35% while the ARIMA model achieved a mean absolute percentage error of 3.29% when predicting one day ahead. Researchers have demonstrated more optimal ways of predicting length of stay and predicting inpatient bed utilization, and this paper demonstrates how the two can be combined to forecast bed needs.

The proposed models have use cases for both short-term and long-term planning. Long-term, perioperative management can forecast when the daily count of surgeries or overnight patient stays will surpass available staffing or equipment. On days of higher need, administrators can limit patient scheduling or acquire more resources. Short term, management can reallocate staff and resources to address anticipated surges in patients. On a more macro level, surgical scheduling could be optimized based on anticipated recovery times and demand for existing hospital beds thus ensuring available beds. As healthcare costs grow and efficient resource utilization is of greater focus, forecasting enabled by this approach to modeling may become an increasingly important component of perioperative management [26–28]. The analytics platforms already utilized in many facilities to better care for patients and minimize resource costs may be further buttressed by such models acting as one more layer of optimization to drive down medical costs in the long run [29]. Future work will explore additional time series models and will include datasets spanning longer time frames.

Interestingly, the difference in model performances in regard to RMSE for predicting bed utilization at 2 weeks and “>2 weeks” were similar. Intuitively, the further out a prediction is made would likely demonstrate poorer performance. We did not observe this finding. This is likely related to the characteristics of the surgical patterns observed in this dataset. The dataset is relatively short, with 42 weeks for training and 10 weeks for testing. In this time, our institution did not observe notable shocks to the system - such as a surge from a pandemic. Instead, we observed a stable trend during this period, thus predictions far out in advance may have similar performance with tasks closer to the index data (e.g. 2 weeks versus “>2 weeks”). For a stable trend, a model making a linear prediction will do better than a prediction from a more complex model. The next step is to test the models against a much longer period of performance that contains a shock. That would give true grounds comparison between the “2 weeks” and “>2 weeks” models.

A major limitation of this study is the single institution design. However, the purpose of the study was not to create a generalizable model to fit any institutional practice as that would be (for now) challenging due to the variation in culture, surgical practices, and patient population across different institutions. Furthermore, practices within a single institution may likely change over time. Previous research into hospital readmission modeling draws similar conclusions regarding the lack of generalizability of these models to other hospital populations [30, 31]. Thus, our objective was to describe the architecture of a machine learning pipeline that may be used to learn a hospital’s patterns for length of stay and surgical volume to then predict its own surgical bed needs in the future. Different institutions may adopt this framework to fit their own data in a customized fashion. Furthermore, even within a single institution, this model would likely need to updated routinely to capture changes the occur over time. We recognize that one year of data is not long enough to make long term conclusions about model longevity. Trends tend to change drastically over many years, which we are not able to observe due to the limited time span. In addition, some of the data such as ASA PS scores and estimated case duration were missing from the dataset and were thus imputed. This may have limited some of the accuracy of the model. We may be able to improve bed utilization forecasts by improving predictions of length of stay and surgery counts, which in turn will be substantially improved with many more years of data.

In conclusion, we demonstrated a pipeline for analyzing features from medical data and using them to make a variety of meaningful forecasts in the perioperative space. Combining comorbidity data and daily surgery counts presents a promising methodology for predicting length of stay and inpatient bed utilization. We hope that with continued and refined usage, this model design may become even more accurate and useful. It is through such emerging technologies that allocation of limited resources like inpatient beds may become more efficient and allow finite hospital resources to be directed to areas of greatest need. Use of this technology in the future may be applied to optimize choke points of work flow in the perioperative area as well as hospital-wide. By integrating these models into the hospital infrastructure, the models can continue to improve over time. Hospital management can monitor resource usage, position personnel ahead of time, and make data-driven purchase decisions with dynamic resources and maximize usage for all stakeholders.

## Data Availability

De-identified data is available upon appropriate data use agreement arrangements with requestor and UC San Diego.
